# Evaluating the independent influence of sexual transmission on HBV infection in China: a modeling study

**DOI:** 10.1186/s12889-021-10408-5

**Published:** 2021-02-19

**Authors:** Miaolei Li, Jian Zu, Mingwang Shen, Guihua Zhuang, Siyuan Chen, Fuzhen Wang, Hui Zheng, Guomin Zhang

**Affiliations:** 1 School of Mathematics and Statistics, Xi’an Jiaotong University, Xi’an, Shaanxi 710049 P. R. China; 2 School of Public Health, Health Science Center, Xi’an Jiaotong University, Xi’an, Shaanxi 710061 P. R. China; 3 Department of Statistics and Actuarial Science, Simon Fraser University, V5A1S6, Burnaby, British Columbia Canada; 4 Chinese Center for Disease Control and Prevention, Beijing, 102206 P. R. China

**Keywords:** Hepatitis B, HBsAg prevalence, Sexual transmission, Age-structured model, Condom promotion

## Abstract

**Background:**

The long-term impact of sexual transmission on the hepatitis B virus (HBV) infection in China remains unclear. This study aims to estimate the independent influence of sexual transmission on HBV infection.

**Methods:**

Based on the natural history of HBV infection and three national serosurvey data of hepatitis B in China, we developed an age- and sex-specific discrete model to describe the transmission dynamics of HBV. The initial conditions of the model were determined according to the age- and sex-specific national serosurvey data in 1992. Based on the national survey data of hepatitis B in 1992 and 2006, by using the Markov Chain Monte Carlo (MCMC) method, we estimated the age- and sex-specific seroclearance rates of hepatitis B surface antigen (HBsAg) and the horizontal transmission rates as well as their 95% confidence intervals (CI). Then we used the age- and sex-specific national serosurvey data of hepatitis B in 2014 to test the accuracy of our model-based estimation. Finally, we evaluated the independent impact of sexual transmission on HBV infection and discussed the long-term effect of promotion of condom use in China.

**Results:**

We estimated that the annual rates of HBsAg seroclearance for males and females aged 1–59 years were respectively 1.04% (95% CI, 0.49–1.59%) and 1.92% (95% CI, 1.11–2.73%). Due to sexual transmission, in 2014, the total number of chronic HBV infections in people aged 0–100 years increased 292,581, of which males increased 189,200 and females increased 103,381. In 2006, the acute HBV infections due to sexual transmission accounted for 24.76% (male: 31.33%, female: 17.94%) and in 2014, which accounted for 34.59% (male: 42.93%, female: 25.73%). However, if the condom usage rate was increased by 10% annually starting in 2019, then compared with current practice, the total number of acute HBV infections from 2019 to 2035 would be reduced by 16.68% (male: 21.49%, female: 11.93%). The HBsAg prevalence in people aged 1–59 years in 2035 would be reduced to 2.01% (male: 2.40%, female: 1.58%).

**Conclusions:**

Sexual transmission has become the predominant route of acute HBV infection in China, especially for men. The promotion of condom use plays a significant role in reducing the cases of acute HBV infection.

**Supplementary Information:**

The online version contains supplementary material available at 10.1186/s12889-021-10408-5.

## Introduction

Hepatitis B virus (HBV) infection remains a serious public health problem in the world and China. People can be infected with HBV through infectious blood, perinatal infection and sexual contact [1]. In order to reduce the HBV infection, China government has implemented hepatitis B vaccination for newborns, but it is still not enough to achieve the goal set by the WHO of eliminating viral hepatitis by 2030 [2].

Several studies have shown that sexual transmission has become the most common route of acute HBV infection among unvaccinated adults in the world [3–7]. Through follow-up investigation and comparative analysis, Hou et al. found that heterosexual contact was the main way of HBV transmission among adults in Taiwan [3]. Sun found that the spouses of HBsAg positive patients had a higher positive rate of HBsAg than that of controls (13.21% vs 6.29%) [4]. Inoue and Hou et al. reported that heterosexual transmission played an increasing role in HBV infection [5–7]. However, the long-term impact of sexual transmission on the HBV infection in China remains unclear.

To describe the transmission dynamics of HBV infection, several mathematical models considering the sexual transmission of HBV have been established [8–11]. Williams et al. studied the heterosexual and homosexual transmission of HBV, and assessed the costs and benefits of different vaccination strategies in the UK [9]. Zou et al. proposed a sex-specific compartmental model to investigate the impact of heterosexual transmission in China, and obtained a basic reproduction number of approximately 2.406 [10]. However, few studies have combined with the recent three serosurvey data of hepatitis B in China.

Moreover, condoms have been shown to play an important role in the prevention of sexually transmitted diseases [5, 12]. However, the survey data of the National Population and Family Planning Commission showed the condom usage rate in China was very low [13]. Up to now, few studies have assessed the role that condoms play in reducing the prevalence of acute HBV infection in China.

Hence, this study aims to evaluate the independent impact of sexual transmission and to predict the long-term effect of promotion of condom use in China. Overall, we developed a sex- and age-specific discrete model and used the Markov Chain Monte Carlo (MCMC) method to assess the independent impact of sexual transmission on the acute HBV infections and HBV-related deaths from 1993 to 2014. Moreover, we predicted the effects of two different condom use strategies on reduction of acute HBV infections and HBV-related deaths in China from 2019 to 2035.

## Material and methods

### Survey data and mathematical model

**Fig. 1:**
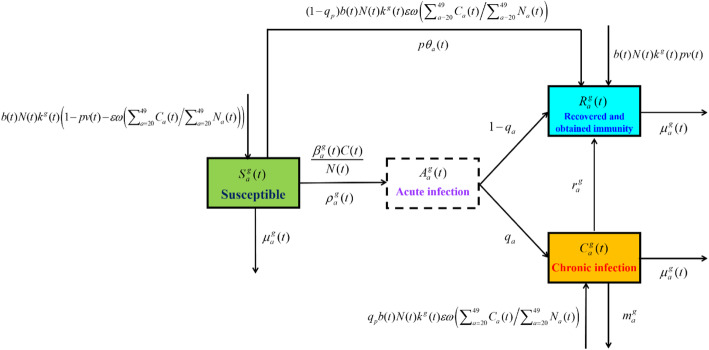
Flow chart of the HBV transmission model. The total population was divided into three compartments: Susceptible individuals (${S}_a^g(t)$), Chronic HBV infections (${C}_a^g(t)$), and Recovered cases (${R}_a^g(t)$). The acutely infectious individuals (${A}_a^g(t)$) were considered to be in a transient process. The force of infection involved three transmission patterns: sexual transmission, perinatal infection and blood transmission

The data used in this paper were derived from three national serosurveys of hepatitis B in China (Table 1) [2, 14, 15]. The demographic data were collected from the National Bureau of Statistics of China. Based on the natural history of HBV infection and the main characteristics of HBV transmission in China, we developed a sex- and age-specific discrete model to describe the transmission dynamics of HBV. Figure 1 showed the structure of the compartment model. In the model, we divided the total population into three compartments: Susceptible individuals; Chronic HBV infections and Recovered cases who had recovered from HBV infection or had Hepatitis B vaccination. Particularly, we considered three different infection routes: one was transmission through sexual contacts, the second was perinatal infection and the third was transmission through contact with infected blood (a detailed model description was provided in the **Supplementary Material**). The initial conditions of the model were determined according to the age- and sex-specific national serosurvey data in 1992. Based on the age- and sex-specific national survey data of hepatitis B in 1992 and 2006, by using the MCMC method, we estimated the age- and sex-specific annual rates of HBsAg seroclearance and the sex-specific horizontal transmission rate, as well as their 95% confidence intervals (Table 2). The detailed description of other parameters and variables were summarized in **Tables S****1****, S****2** and **Figure** **S1**.

**Table 1:** Age- and sex-specific prevalence of HBsAg in 1992, 2006 and 2014 in China

Age group (years)	Male (%)			Female (%)			Total (%)		
	1992	2006	2014	1992	2006	2014	1992	2006	2014
1–4	10.68	1.21	0.40	8.47	0.93	0.35	9.67	1.08	0.38
5–9	12.06	1.82	0.64	8.16	1.37	0.87	10.22	1.61	0.75
10–14	12.74	3.77	1.30	9.65	2.95	1.16	11.27	3.37	1.23
15–19	11.41	8.48	2.19	9.28	5.79	1.72	10.35	7.17	1.95
20–24	11.20	8.89	5.08	8.08	7.67	4.19	9.49	8.29	4.57
25–29	11.64	10.76	5.61	8.10	6.65	4.69	9.61	8.76	5.06
30–34	12.46	9.93	–	9.12	6.58	–	10.64	8.33	–
35–39	11.22	10.46	–	7.50	6.60	–	9.22	8.59	–
40–49	10.83	10.47	–	7.90	6.85	–	9.31	8.75	–
50–59	8.55	9.23	–	6.67	7.04	–	7.58	8.20	–
Total	**11.33**	**7.55**	**1.59**	**8.23**	**5.32**	**1.67**	**9.75**	**6.47**	**1.63**

**Note.** “--” means that it was not covered in the survey

**Table 2:** Estimated age- and sex-specific annual rate of HBsAg seroclearance

Age group (years)	Male		Female	
	Annual rate (%)	95% CI	Annual rate (%)	95%CI
1–4	1.04	(0.00, 2.36)	19.47	(16.33, 22.61)
5–9	5.45	(2.66, 8.24)	4.62	(2.11, 7.13)
10–14	1.14	(0.00, 2.93)	0.68	(0.00, 1.60)
15–19	1.35	(0.01, 2.68)	0.78	(0.00, 1.96)
20–24	2.10	(0.62, 3.58)	4.41	(2.97, 5.85)
25–29	0.60	(0.00, 1.48)	1.20	(0.00, 2.72)
30–34	0.91	(0.00, 2.00)	1.87	(0.00, 4.22)
35–39	0.98	(0.00, 2.16)	0.91	(0.00, 2.46)
40–49	1.16	(0.00, 2.62)	1.81	(0.39, 3.23)
50–59	2.02	(0.16, 3.88)	1.32	(0.04, 2.61)
Total	**1.04**	**(0.49, 1.59)**	**1.92**	**(1.11, 2.73)**

**Note.** If the lower bound of the confidence interval was negative, then we set it to 0.00

### Model calibration

We calibrated and validated our model based on the national serosurvey data of hepatitis B in 2006 and 2014. More specifically, we substituted the estimated parameter values into the model, after 14 iterations, we obtained the estimated prevalence of HBsAg in males and females aged 1–59 years in 2006. By comparing with the national serosurvey data of hepatitis B in 2006, we can see that the serosurvey data were basically within the 95% confidence intervals and the largest errors in males and females was 1.04 and 0.91%, respectively, which indicated that the estimated prevalence of HBsAg fitted well with the serosurvey data of hepatitis B in 2006 (see **Figure** **S2****a,b**). Then we substituted the estimated parameter values into the model, after 22 iterations, we obtained the estimated prevalence of HBsAg in males and females aged 1–29 years in 2014. We can see that the estimated prevalence of HBsAg in males and females also fitted well with the serosurvey data of hepatitis B in 2014 (see **Figure** **S2****c,d**). Therefore, our model-based estimation were credible for evaluating the independent impact of sexual transmission on HBV infection and predicting the long-term effect of promotion of condom use in China in the future.

### Impact of sexual transmission

To evaluate the impact of sexual transmission, we estimated the sex-specific prevalence of HBsAg in the population aged 1–59 years from 1993 to 2014, the sex-specific number of acute HBV infections and HBV-related deaths with or without considering sexual transmission. Moreover, we estimated the increase in HBsAg prevalence due to sexual transmission for people aged 1–59 years in 2006 and 2014, as well as the proportion of sex-specific acute HBV infections and HBV-related deaths due to sexual transmission in 2006 and 2014.

### Condom use strategy

We accessed two different condom use strategies: (i) the condom usage rate reached 100% starting in 2019; and (ii) the condom usage rate was increased by 10% annually starting in 2019. We compared the two strategies with the current practice by numerical simulation. Particularly, we predicted the total number of sex-specific chronic HBV infections, acute HBV infections and HBV-related deaths, as well as the corresponding number of sex-specific chronic HBV infections, acute HBV infections and HBV-related deaths in each age group in China from 2019 to 2035 under the two different condom use strategies.

### Sensitivity analysis

The sensitivity analysis was performed from two aspects. First, we assessed which parameters would affect the estimated age- and sex-specific annual rates of HBsAg seroclearance. Second, we identified which parameters would affect the predictions of chronic HBV infections, acute HBV infections and HBV-related deaths of males and females in 2035. Specifically, the initial horizontal transmission rate ${\beta}_a^g(1992)$, the vaccination protection rate *p* and the perinatal infection rate *ε* were assessed. Here, we assumed that each parameter changed in terms of their upper and lower limit values and that the other parameters remained unchanged.

## Results

### Increase in the number of chronic HBV infections due to sexual transmission

**Fig. 2:**
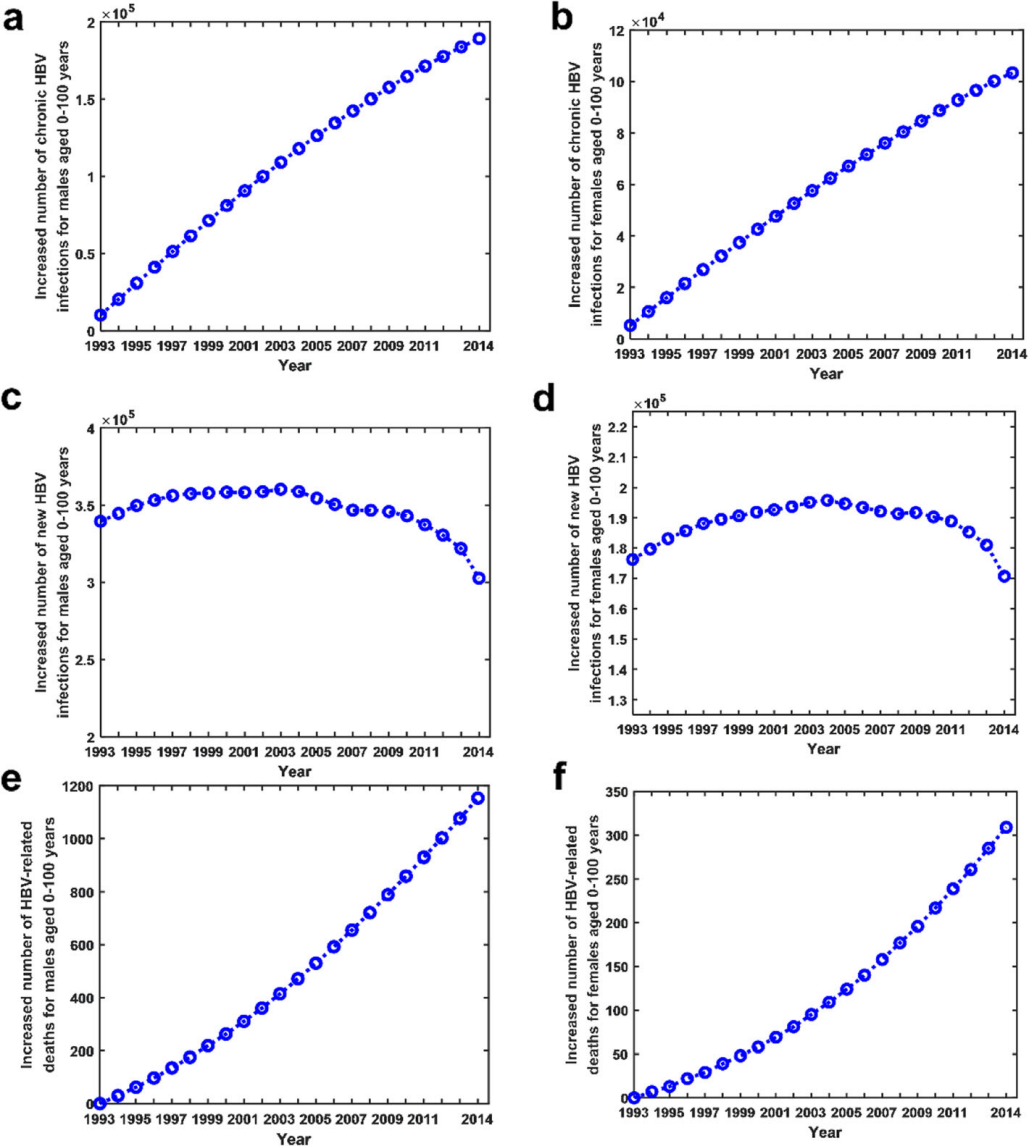
Impact of sexual transmission. **a** Increased number of chronic HBV infections for males aged 0–100 years in China from 1993 to 2014. **b** Increased number of chronic HBV infections for females aged 0–100 years in China from 1993 to 2014. **c** Increased number of acute HBV infections for males aged 0–100 years in China from 1993 to 2014. **d** Increased number of acute HBV infections for females aged 0–100 years in China from 1993 to 2014. **e** Increased number of HBV-related deaths for males aged 0–100 years in China from 1993 to 2014. (f) Increased number of HBV-related deaths for females aged 0–100 years in China from 1993 to 2014

The results showed that due to sexual transmission, the total number of chronic HBV infections in people aged 0–100 years in China increased 1,598,320 from 1993 to 2006 and 3,658,439 from 1993 to 2014. Particularly, the total number of chronic HBV infections in people aged 0–100 years due to sexual transmission increased 292,581 in 2014, of which males increased 189,200 and females increased 103,381 (see Fig. 2a,b). Furthermore, we found that sexual transmission had a more important impact on chronic HBV infections in males in China than in females.

### Increase in the number of acute HBV infections due to sexual transmission

The results showed that due to sexual transmission, the total number of acute HBV infections in people aged 0–100 years in China increased by 12.82% from 1993 to 2006 and 17.22% from 1993 to 2014. Particularly, among the total number of acute HBV infections in people aged 0–100 years in 2014, new infections due to sexual transmission accounted for 34.59% (=473,462/1368805, where the numerator 473,462 was the number of acute HBV infections due to sexual transmission in 2014, the denominator 1,368,805 was the number of acute HBV infections in people aged 0–100 years in 2014), of which males accounted for 42.93% (=302,737/705171) and females accounted for 25.73% (=170,725/663634) (see Fig. 2c,d). Furthermore, we found that sexual transmission had a more important impact on acute HBV infections in males in China than in females.

### Increase in the number of HBV-related deaths due to sexual transmission

We estimated that due to sexual transmission, the number of HBV-related deaths of people aged 0–100 years in China increased by 0.10% from 1993 to 2006 and 0.19% from 1993 to 2014. Particularly, among the total number of HBV-related deaths of people aged 0–100 years in 2014, HBV-related deaths due to sexual transmission accounted for 0.38% (=1462/382845), of which males accounted for 0.42% (=1153/273670) and females accounted for 0.28% (=309/109175) (see Fig. 2e,f). Thus, the impact of sexual transmission on HBV-related deaths would gradually increase over time.

### Reduction of chronic HBV infections due to condom use

**Fig. 3:**
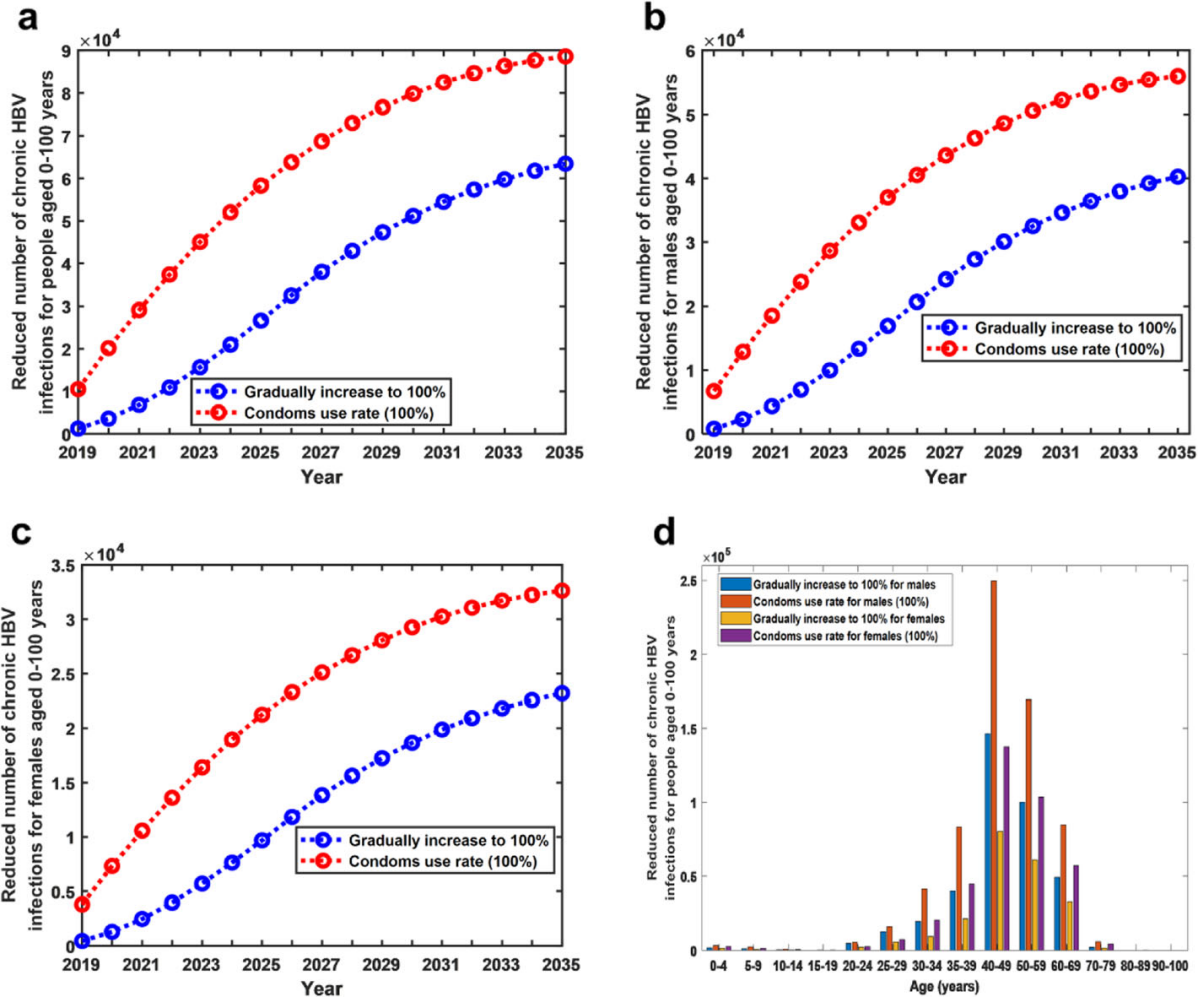
Reduction of chronic HBV infections under two different condom strategies. **a** Reduced number of chronic HBV infections for people aged 0–100 years under two different condom strategies. **b** Reduced number of chronic HBV infections for males aged 0–100 years under two different condom strategies. **c** Reduced number of chronic HBV infections for females aged 0–100 years under two different condom strategies. **d** Reduced number of chronic HBV infections for males and females in each age group from 2019 to 2035 under two different condom strategies

The results showed that a higher condom usage rate resulted in a significant reduction in the number of chronic HBV infections in both males and females. Under the current practice, the total number of chronic HBV infections in people aged 0–100 years from 2019 to 2035 is 863,550,362, of which infections in males is 514,551,487 and in females is 348,998,875. In comparison with the current practice, if the condom usage rate was increased by 10% annually starting in 2019, the total number of chronic HBV infections in people aged 0–100 years from 2019 to 2035 would be reduced by 0.07% (863,550,362 vs 862,955,659), of which infections in males would be reduced by 0.07% (514,551,487 vs 514,173,630) and in females by 0.06% (348,998,875 vs 348,782,029) (see Table 3). However, if the condom usage rate reached 100% starting in 2019, the number of chronic HBV infections in people aged 0–100 years from 2019 to 2035 would be reduced by 0.12% (863,550,362 vs 862,505,969), of which infections in males would be reduced by 0.13% (514,551,487 vs 513,889,292) and in females by 0.11% (348,998,875 vs 348,616,677) (see Fig. 3a,b,c). Particularly, in 2035, the number of chronic HBV infections in people aged 0–100 years would be reduced by 0.23% (38,820,497 vs 38,731,913), of which infections in males would be reduced by 0.25% (22,806,441 vs 22,750,481) and in females by 0.20% (16,014,056 vs 15,981,432). The HBsAg prevalence in people aged 1–59 years in 2035 would be reduced to 2.01%, of which the prevalence in males would be reduced to 2.40% and in females to 1.58%.

**Table 3:** Numbers of sex-specific chronic HBV infections, acute HBV infections and HBV-related deaths under two different condom use strategies

Condom use strategies	Number of chronic HBV infections in males from 2019 to 2035	Number of chronic HBV infections in females from 2019 to 2035	Number of acute HBV infections in males from 2019 to 2035	Number of acute HBV infections in females from 2019 to 2035	Number of HBV-related deaths of males from 2019 to 2035	Number of HBV-related deaths of females from 2019 to 2035
Current practice	514,551,487	348,998,875	745,467	7,534,591	5,268,115	2,266,011
Condom usage rate was increased by 10% annually starting in 2019	514,173,630	348,782,029	5,852,870	6,635,811	5,265,513	2,265,244
Decrease from current practice (%)	0.07%	0.06%	21.49%	11.93%	0.05%	0.03%
Condom usage rate reached 100% starting in 2019	513,889,292	348,616,677	5,125,174	6,221,016	5,263,475	2,264,638
Decrease from current practice (%)	0.13%	0.11%	31.25%	17.43%	0.09%	0.06%

In addition, if the condom usage rate was increased by 10% annually starting in 2019, then from 2019 to 2035, in the 40–49 age group, the number of chronic HBV infections in males and females would reduce the most (males:146439, females:80273) (see Fig. 3d).

### Reduction of acute HBV infections due to condom use

**Fig. 4:**
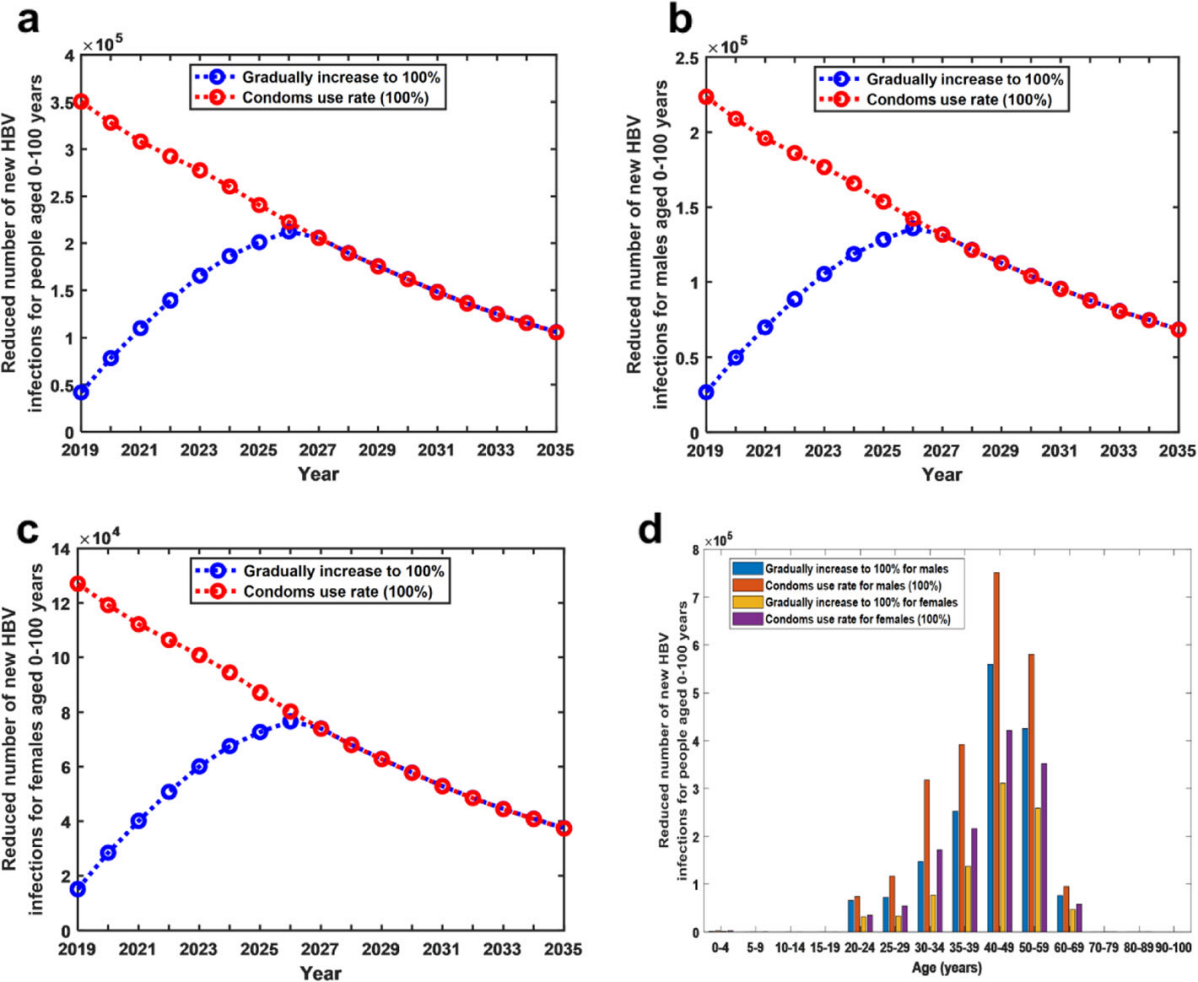
Reduction of acute HBV infections under two different condom strategies. **a** Reduced number of acute HBV infections for people aged 0–100 years under two different condom strategies. **b** Reduced number of acute HBV infections for males aged 0–100 years under two different condom strategies. **c** Reduced number of acute HBV infections for females aged 0–100 years under two different condom strategies. **d** Reduced number of acute HBV infections for males and females in each age group from 2019 to 2035 under two different condom strategies

The results show that the promotion of condom use would play a significant role in reducing acute HBV infections in both males and females. Under the current practice, the total number of acute HBV infections in people aged 0–100 years from 2019 to 2035 is 14,989,270, of which new infections in males is 7,454,679 and in females is 7,534,591. If the condom usage rate was increased by 10% annually starting in 2019, the total number of acute HBV infections in people aged 0–100 years from 2019 to 2035 would be reduced by 16.68% (14,989,270 vs 12,488,681), of which new infections in males would be reduced by 21.49% (7,454,679 vs 5,852,870) and in females by 11.93% (7,534,591 vs 6,635,811). In contrast, if the condom usage rate reached 100% starting in 2019, the number of acute HBV infections in people aged 0–100 years from 2019 to 2035 would be reduced by 24.30% (14,989,270 vs 11,346,190), of which the number in males would be reduced by 31.25% (7,454,679 vs 5,125,174) and in females by 17.43% (7,534,591 vs 6,221,016) (see Fig. 4a,b,c).

In addition, if the condom usage rate was increased by 10% annually starting in 2019, then from 2019 to 2035, in the 40–59 age group, the number of acute HBV infections in males and females would reduce the most (males:559415, females:310901) (see Fig. 4d).

### Reduction of HBV-related deaths due to condom use

**Fig. 5:**
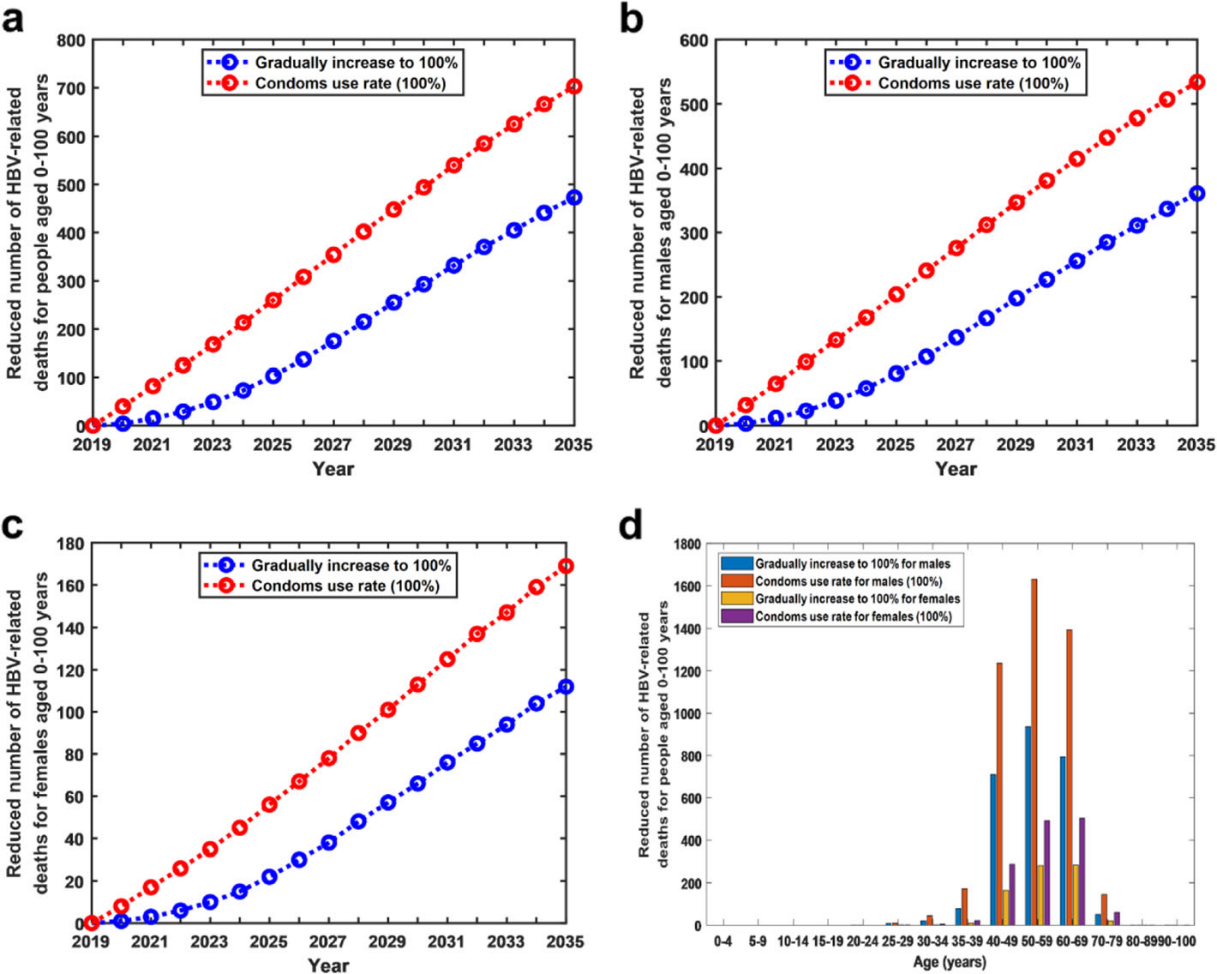
Reduction of HBV-related deaths under two different condom strategies. **a** Reduced number of HBV-related deaths for people aged 0–100 years under two different condom strategies. **b** Reduced number of HBV-related deaths for males aged 0–100 years under two different condom strategies. **c** Reduced number of HBV-related deaths for females aged 0–100 years under two different condom strategies. **d** Reduced number of HBV-related deaths for males and females in each age group from 2019 to 2035 under two different condom strategies

The results showed that a higher condom usage rate resulted in a significant reduction in the number of HBV-related deaths of both males and females. Under the current practice, the total number of HBV-related deaths in people aged 0–100 years from 2019 to 2035 is 7,534,126, of which the HBV-related deaths in males is 5,268,115 and in females is 2,266,011. However, if the condom usage rate was increased by 10% annually starting in 2019, the total number of HBV-related deaths of people aged 0–100 years from 2019 to 2035 would be reduced by 0.04% (7,534,126 vs 7,530,757), of which the number of deaths of males would be reduced by 0.05% (5,268,115 vs 5,265,513) and of females by 0.03% (2,266,011 vs 2,265,244). In contrast, if the condom usage rate reached 100% starting in 2019, the number of HBV-related deaths of people aged 0–100 years from 2019 to 2035 would be reduced by 0.08% (7,534,126 vs 7,528,113), of which the number of deaths of males would be reduced by 0.09% (5,268,115 vs 5,263,475) and of females by 0.06% (2,266,011 vs 2,264,638) (see Fig. 5a,b,c).

In addition, if the condom usage rate reached 100% starting in 2019, then from 2019 to 2035, in the 50–59 age group, the number of HBV-related deaths in males would reduce the most. In the 60–69 age group, the number of HBV-related deaths in females would reduce the most (see Fig. 5d).

### Results of sensitivity analysis

As shown in **Figures** **S3****,** **S4**, the sex-specific annual rate of HBsAg seroclearance would fluctuate only within a small range around the base-case result with the change of any one of the parameters between its upper and lower limit values. Among these parameters, the horizontal transmission rate had some impact on the annual rate of HBsAg seroclearance, the second parameter was the perinatal infection rate.

Moreover, from **Table** **S3**, we can see that the horizontal transmission rate had some impact on the number of chronic HBV infections in 2035. The vaccination protection rate had some impact on the number of acute HBV infections in males and females in 2035. However, the impact on the number of acute HBV infections in males and females in 2035 is small. If the vaccination protection rate took the upper limit value 0.95, then the number of acute HBV infections in males and females in 2035 would decrease by 31.31% (307571vs 211,263) and 31.02% (326,930 vs 225,501), respectively (see **Table** **S3**).

## Discussion

Estimating the independent impact of sexual transmission on HBV infection was of great significance for the prevention and control of hepatitis B all over the world. In this paper, based on the natural history of HBV infection and three national serosurvey data of hepatitis B in China, we developed an age- and sex-specific discrete model at the population level to evaluate the influence of sexual transmission on HBV infection in China. Unlike our previous work [16], the innovations of this study were reflected in the following three aspects. First, we considered the gender differences in HBV infection and estimated the age- and sex-specific annual rate of HBsAg seroclearance by using the Markov Chain Monte Carlo (MCMC) method. Second, we considered a special transmission route of HBV separately, that is, sexual transmission among people aged 20–60 years, and evaluated the independent impact of sexual transmission on HBV infection. Finally, we introduced another important intervention strategy, increasing condom usage rate, and predicted the long-term effect of condom use on reducing the prevalence of hepatitis B in China.

This study showed that sexual transmission had an important impact on acute HBV infections and HBV-related deaths in both males and females, and the impact of sexual transmission would gradually increase over time. These findings were consistent with the results in Hou et al. [7] Hou et al. revealed that heterosexual transmission accounted for an increasing proportion of HBV infections [7]. Besides, our study indicated that among the total number of acute HBV infections in people aged 0–100 years in 2014, new infections due to sexual transmission accounted for 34.59%, of which males accounted for 42.93% and females accounted for 25.73%. This demonstrated that sexual transmission has become the predominant route of acute HBV infections in China. These findings were also consistent with the results in Hou et al. [3] Hou et al. investigated 60 acutely HBV infectious individuals and 93 controls among adults in Taiwan, they found that heterosexual contact was the main way of HBV infection among adults in Taiwan [3]. Moreover, our study also showed that sexual transmission had more impact on males than females in China. This might be due to the higher transmission probability of HBV for males than females, and the underlying mechanisms were worthy to be studied further. These quantitative findings reminded us that in order to prevent and control the transmission of HBV more effectively, special attention should be paid to the influence of sexual transmission on HBV infection.

Furthermore, this study showed that increasing condom usage rate would significantly reduce the total number of acute HBV infections and HBV-related deaths in both males and females, especially in older age groups. The higher the condom usage rate and the longer the time, the greater the decrease in acute HBV infections and HBV-related deaths in both males and females. Particularly, if the condom usage rate reached 100% starting in 2019, then the total number of acute HBV infections from 2019 to 2035 would be reduced by 24.30% (14,989,270 vs 11,346,190), of which males would be reduced by 31.25% (7,454,679 vs 5,125,174) and females by 17.43% (7,534,591 vs 6,221,016). These results were consistent with previous findings in several cohort studies that evaluated the effectiveness of condom use in preventing sexually transmitted infections such as gonorrhoea, chlamydial infection and human immunodeficiency virus (HIV) infections and so on. These study revealed that using condoms for 100% of sex acts was associated with a significant reduction for sexually transmitted infections [17, 18]. However, the condom usage rate in China was very low currently according to the survey data of the National Population and Family Planning Commission [13]. Therefore, we believed that if we could increase the usage rate of condoms as much as possible, then more and more chronic HBV infections, acute HBV infections and HBV-related deaths would be prevented in China.

There were some limitations in this study. First, we focused only on heterosexual transmission and ignored homosexual transmission because of the lack of national survey data. Second, we assumed that the sex-specific horizontal transmission rate decreased exponentially since 1993 with the implementation of prevention and control measures of hepatitis B, but some age groups might be at higher risk for HBV infection because of particular risk factors, such as drug injection. Third, we used the age-specific prevalence of HBV in 1992 as the prevalence of HBV for males and females in 1992 because the sex-specific prevalence of HBV was not assessed in 1992, which might cause some deviations of the estimated results.

In conclusion, this study developed a novel method to quantitatively evaluate the independent impact of sexual transmission on HBV infection in China. This model-based study showed an urgent need for effective intervention in terms of the sexual transmission of hepatitis B in China. In particular, this study revealed that the promotion of condom use played an important role in the prevention of chronic HBV infections, acute HBV infections and HBV-related deaths, and great efforts should be made to increase the condom usage rate among the population aged 20–60 years in China. These findings provided quantitative reference for the prevention and control of hepatitis B in China and other high endemic areas.

## Supplementary Information

**Additional file 1 Supplementary Material.** The supplementary material described in detail the formulation of transmission model and parameter estimation presented in the main text.

**Additional file 2 Supplementary Data.** The supplementary data included initial conditions and estimated parameter values of the models (1) and (2).

## Data Availability

All data generated or analysed during this study are included in this published article and its supplementary information files.
