# Supplementary material for: Evaluating the independent influence of sexual transmission on HBV infection in China: a modeling study
Source: BMC Public Health. 2021 Feb 19;21:388. doi: 10.1186/s12889-021-10408-5 (PMC7893752; doi:10.1186/s12889-021-10408-5)
Supplement: Supplementary file 1 — Additional file 1 Supplementary Material. The supplementary material described in detail the formulation of transmission model and parameter estimation presented in the main text. [file 12889_2021_10408_MOESM1_ESM.docx]

**Supplementary Material**

**Evaluating the independent influence of sexual transmission on HBV infection in China: A modeling study**

**Miaolei Li^1^, Jian Zu^1,*^, Mingwang Shen^2^, Guihua Zhuang^2^, Siyuan Chen^3^, Fuzhen Wang^4^, Hui Zheng^4^, Guomin Zhang^4,*^**

**^1^** School of Mathematics and Statistics, Xi’an Jiaotong University, Xi’an, Shaanxi, 710049, P. R. China

**^2^** School of Public Health, Health Science Center, Xi’an Jiaotong University, Xi’an, Shaanxi, 710061, P. R. China

**^3^** Department of Statistics and Actuarial Science, Simon Fraser University, Burnaby, British Columbia, V5A1S6, Canada

**^4^** Chinese Center for Disease Control and Prevention, Beijing, 102206, P. R. China

**^*^Corresponding author:** Jian Zu, PhD, School of Mathematics and Statistics, Xi’an Jiaotong University, Xi’an, Shaanxi, 710049, P. R. China (Email address: [jianzu@xjtu.edu.cn](mailto:jianzu@xjtu.edu.cn)).

This is a supplementary material describing in detail the formulation of transmission model and parameter estimation presented in the main text.

**1. Data sources and mathematical model**

We collected the reported data from the national serosurveys of hepatitis B in 1992, 2006 and 2014 in China (**Table 1**).^1-3^ Three national serosurveys were conducted in 160 disease surveillance sites in 31 provinces and cities nationwide, and the multi-stage random sampling method was used to extract the data of the resident population. In 1992, the data of a total of 61,702 people (29,693 males and 32,009 females) aged 1-59 years were investigated, and the prevalence of HBsAg in males was higher than that in females (11.33% vs 8.23%).^2^ In 2006, the data of a total of 81,775 people (38,895 males and 42,880 females) aged 1-59 years were investigated, and the results from this survey showed that the prevalence of HBsAg in males and females was lower than that in 1992.^1^ In 2014, the data of only 31,713 people (15,814 males and 15,899 females) aged 1-29 years were investigated to assess the effect of the hepatitis B vaccination in newborns, and the results showed that the prevalence of HBsAg in both males and females was reduced significantly compared with 1992 and 2006 ( see **Table 1**).^3^

Based on the natural history of the HBV infection and the main characteristics of HBV transmission in China, we proposed an age-structured discrete model by further considering another important transmission route for hepatitis B, sexual transmission among adults in the sexually active age classes and another important intervention, the promotion of condom use.^1,4-7^ Specifically, we divided the total population into three compartments: susceptible$S_{a}^{g}(t)$; chronic HBV infections$C_{a}^{g}(t)$; and individuals who had recovered from HBV infection and obtained immunity$R_{a}^{g}(t)$, where $g=m,f$denoted the group of males or females, $a$ denoted the age, and $t$ denoted the time. Here, we selected “year” as the basic unit of time. The acutely infectious individuals $A_{a}^{g}(t)$were considered to be in a transient process given the short average duration of 3 months compared with years for the chronic period.^8,9^ Particularly, apart from perinatal infection from infected mothers to their infants and horizontal transmission (non-sexual transmission) among the whole population, in this study, we considered sexual transmission among adults in the sexually active age classes. For simplicity, we made the following assumptions:

1. We only focused on heterosexual transmission.
2. The whole population was homogeneously distributed.
3. The maximum age of people was 100 years old, and the whole population was divided into 101 age groups.
4. Sexual activities were considered to begin at age 20 and continue until age 60 for both males and females in order to facilitate the direct use of data derived from the national serosurveys of hepatitis B in 1992, 2006 and 2014 in China.
5. Susceptible individuals aged 1-100 years could be infected by contact with all age groups of infectious individuals, and the transmission rate in same age groups due to horizontal transmission among individuals of the same sex was assumed to be the same.

Based on the above assumptions, the age-structured compartmental model was described by equations (1) and (2).

For individuals aged 0 year ($0\leq a<1$):

$\left\{\begin{matrix} S_{0}^{g}(t+1)=b(t)N(t)k^{g}(t)\left(1-pv(t)-\varepsilon\omega\left(\sum_{a=20}^{49} C_{a}(t)/\sum_{a=20}^{49} N_{a}(t)\right)\right), \\ C_{0}^{g}(t+1)=q_{p}b(t)N(t)k^{g}(t)\varepsilon\omega\left(\sum_{a=20}^{49} C_{a}(t)/\sum_{a=20}^{49} N_{a}(t)\right), \\ R_{0}^{g}(t+1)=b(t)N(t)k^{g}(t)pv(t)+(1-q_{p})b(t)N(t)k^{g}(t)\varepsilon\omega\left(\sum_{a=20}^{49} C_{a}(t)/\sum_{a=20}^{49} N_{a}(t)\right). \end{matrix}\right.$ (1)

For individuals aged 1-100 years:

$\left\{\begin{matrix} S_{a+1}^{g}(t+1)=\left(1-\mu_{a}^{g}(t)\right)S_{a}^{g}(t)-p\theta_{a}(t)S_{a}^{g}(t)-\lambda_{a}^{g}(t)S_{a}^{g}(t), \\ C_{a+1}^{g}(t+1)=\left(1-\mu_{a}^{g}(t)\right)C_{a}^{g}(t)+q_{a}\lambda_{a}^{g}(t)S_{a}^{g}(t)-m_{a}^{g}C_{a}^{g}(t)-r_{a}^{g}C_{a}^{g}(t), \\ R_{a+1}^{g}(t+1)=\left(1-\mu_{a}^{g}(t)\right)R_{a}^{g}(t)+p\theta_{a}(t)S_{a}^{g}(t)+(1-q_{a})\lambda_{a}^{g}(t)S_{a}^{g}(t)+r_{a}^{g}C_{a}^{g}(t). \end{matrix}\right.$(2)

The force of infection $\lambda_{a}^{g}(t)$ for the population aged 1-100 years was given by

$\lambda_{a}^{g}(t)=\left\{\begin{matrix} \frac{\beta_{a}^{g}(t)C(t)}{N(t)}, 1\leq a<20, 60<a\leq100, \\ \frac{\beta_{a}^{g}(t)C(t)}{N(t)}+\rho_{a}^{g}(t), 20\leq a\leq60. \end{matrix}\right.$ (3)

where $C(t)$denotes the total number of chronic HBV infections, $N(t)$denotes the total population, $\frac{\beta_{a}^{g}(t)C(t)}{N(t)}$denotes the force of infection due to horizontal transmission, and $\beta_{a}^{g}(t)$represents the sex-specific horizontal transmission rate for susceptible individuals in age group $a$ in year $t$. The force of infection due to sexual transmission was described as

$\rho_{a}^{m}(t)=(1-\pi(t))\beta_{m}c_{a}^{m}\frac{\sum_{a=20}^{60} C_{a}^{f}(t)}{\sum_{a=20}^{60} N_{a}^{f}(t)}$ (4)

$\rho_{a}^{f}(t)=(1-\pi(t))\beta_{f}c_{a}^{f}\frac{\sum_{a=20}^{60} C_{a}^{m}(t)}{\sum_{a=20}^{60} N_{a}^{m}(t)}$ (5)

where $\beta_{m}$and $\beta_{f}$ represent the sexual transmission probabilities of HBV per-partnership for males and females, $c_{a}^{m}$and $c_{a}^{f}$denote the average numbers of sexual partners per year for males and females, and $\pi(t)$denotes the condom use rate for males and females in year $t$. For simplicity, we divided the individuals aged 20 to 60 years into 6 groups (20-24, 25-29, 30-34, 35-39, 40-49 and 50-60 years), and in each group, the average numbers of sexual partners per year and the initial horizontal transmission rate for males and females were assumed to be the same. The total number of sexual partners for males per year should be equal to the total number of sexual partners for females per year, that is,

$\sum_{a=20}^{60} c_{i}^{m}N_{a}^{m}(t)=\sum_{a=20}^{60} c_{i}^{f}N_{a}^{f}(t)$ , $i=1, 2 ,..., 6$ (6)

where $c_{i}^{m}$and $c_{i}^{f}$denote the average numbers of sexual partners per year in each age group for males and females, and $c_{i}^{m}$and $c_{i}^{f}$ are assumed to remain the same every year starting in 1992. In this model, acute HBV infections come from perinatal infection, horizontal transmission and sexual transmission.

**2. Initial conditions of models (1) and (2)**

The initial time of equations (1) and (2) was taken as$t_{0}=1992$. The age-specific and sex-specific total population number$N_{a}^{g}(1992)$equalled the age-specific and sex-specific proportion of newborns in 1990 multiplied by the calibrated age-specific total population number in 1992. The age-specific and sex-specific chronic HBV infections$C_{a}^{g}(1992)$equalled the age-specific and sex-specific prevalence rate of HBsAg in 1992 multiplied by the age-specific and sex-specific total population number (see **Supplementary data.xlsx, columns 5 and 6**). The individuals who recovered from HBV infection and obtained immunity$R_{a}^{g}(1992)$equalled the difference between the age-specific and sex-specific prevalence of HBV and HBsAg in 1992 multiplied by the age-specific and sex-specific chronic HBV infections (see **Supplementary data.xlsx, columns 7 and 8**). The sex-specific prevalence of HBV was not assessed in 1992; here, we used the age-specific prevalence of HBV in 1992 as the prevalence of HBV for males and females in 1992. The susceptible individuals $S_{a}^{g}(1992)$(see **Supplementary data.xlsx, columns 3 and 4**) were calculated based on the following equation:

$$S_{a}^{g}(1992)=N_{a}^{g}(1992)-C_{a}^{g}(1992)-R_{a}^{g}(1992).$$

**3. Estimation of model input parameters**

Next, we determined the parameter values in models (1) and (2). First, some of the parameters were derived from published literature and nationally reported data. The birth rate $b(t)$and the sex-specific proportion of newborns $k^{g}(t)$were determined according to the National Bureau of Statistics of China, $b(t)=b(2016), \text{（}(t>2016)$and$k^{g}(t)=k^{g}(2015)\text{，}, (t>2015)$(see **Figure S1a**).^10-12^ The vaccination coverage rate for newborns$v(t)$was obtained from the National Immunization Survey, $v(t)=v(2014), \text{（}(t>2014)$.^13^ The proportion of HBsAg-positive mothers aged 20-49 years in the total prevalence of HBsAg $\omega$was calculated by dividing the proportion of HBsAg-positive mothers among females by the proportion of HBsAg-positive individuals among the population aged 20-49 years. The age-specific and sex-specific mortality rates of HBV-related diseases $m_{a}^{g}$were calculated by dividing the age-specific and sex-specific total number of HBV-related cirrhosis and HCC by the age-specific and sex-specific number of chronic HBV infections (see **Figure S1b**).^14^ Here, we assumed that the sex-specific number of chronic HBV infections was calculated based on the sex-specific prevalence rate of HBsAg in 2006. The age-specific and sex-specific total death rate$d_{a}^{g}(t)$was obtained according to statistics released by the National Bureau of Statistics in 1995, 2000, 2005, 2010 and 2015 (see **Figure S1c**, **Figure S1d**).^15,16^ Hence, the age-specific and sex-specific death rate of non-HBV-related diseases$\mu_{a}^{g}(t)$was determined based on the equation $\mu_{a}^{g}(t)=(d_{a}^{g}(t)N_{a}^{g}(t)-m_{a}^{g}C_{a}^{g}(t))/N_{a}^{g}(t)$.^16^ The condom use rates for males and females $\pi(t)$ were determined from the China Population and Employment Statistics Yearbook (1993-2016) and $\pi(t)=\pi(2016)\text{，}, (t>2016)$(see **Supplementary data.xlsx, column 9**) .^15,17^

Second, we estimated the initial horizontal transmission rate$\beta_{a}^{g}(1992)$and the average numbers of sexual partners per year for males and females in each age group. To estimate the initial horizontal transmission rate$\beta_{a}^{g}(1992)$, we first estimated the initial force of HBV infection $\lambda_{a}^{g}(1992)$by using a classical catalytic model to describe the natural process of HBV infection, similar to our previous work.^16,18^ Because there were no sex-specific prevalence of HBV for males and females in 1992, we used the total prevalence of HBV in 1992 instead, and we selected the same exponential function to fit the reported prevalence data, that is,

$\lambda_{a}^{g}(1992)=b_{1}+b_{2}\exp(b_{3}a)$.

By using the Markov Chain Monte Carlo (MCMC) method, we obtained $b_{1}=0.0124$, $b_{2}=0.2701$ and $b_{3}=\text{-}0.4618$, and the corresponding 95% confidence intervals were (0.0107, 0.0141), (0.2107, 0.3295) and (-0.5870, -0.3366), respectively. Based on the first formula in equation (3), we obtained the initial horizontal transmission rate $\beta_{a}^{g}(1992)=\lambda_{a}^{g}(1992)N(1992)/C(1992)$for 1≤*a*<20 and 60<*a*≤100. Next, we estimated $\beta_{a}^{g}(1992)$and the average numbers of sexual partners per year for males and females for 20≤*a*≤60 simultaneously. In the above analyses, we divided the individuals aged 20 to 60 years into 6 groups (20-24, 25-29, 30-34, 35-39, 40-49 and 50-60 years), and we assumed that $\beta_{a}^{g}(1992)$and the average numbers of sexual partners per year for males and females were the same in each age group. Specifically, we chose the above exponential function for $\lambda_{a}^{g}(1992)$as the observation function. The initial values in each group were selected according to the sexual behaviour and lifestyle in China, and we assumed an approximate 1:1 proportional relationship between horizontal and sexual transmission.^19,20^ By using the piecewise fitting method, we obtained the initial horizontal transmission rate $\beta_{a}^{g}(1992)$and its 95% confidence intervals (see **Figure S1e**, **Figure S1f**), as well as the average numbers of sexual partners per year for males and females in each age group (see **Table S2**).

Finally, we estimated the sex-specific annual rate of HBsAg seroclearance $r_{a}^{g}$ and the sex-specific horizontal transmission rate $\beta_{a}^{g}(t)$. For simplicity, we divided the individuals aged 1-59 years into 10 groups (1-4, 5-9, 10-14, 15-19, 20-24, 25-29, 30-34, 35-39, 40-49 and 50-59 years), and in each group, the age-specific and sex-specific annual rates of HBsAg seroclearance were assumed to be different. The sex-specific annual rate of HBsAg seroclearance for individuals aged 60-100 years was assumed to be 0.015.^16^ In addition, we assumed that the sex-specific horizontal transmission rate$\beta_{a}^{g}(t)$decreased exponentially with the implementation of prevention and control measures of hepatitis B.^3,21,22^ After determining the initial conditions and model input parameters, based on the sex-specific prevalence of HBsAg in populations aged 1-59 years in 2006, we used the MCMC method to estimate the age- and sex-specific annual rates of HBsAg seroclearance $r_{a}^{g}$and the corresponding 95% confidence intervals by setting the iteration number to 10,000 and the first 8,000 times as burn-in periods. The results showed that the average annual rate of HBsAg seroclearance for males aged 1-59 years was 1.04% (95% CI, 0.49-1.59%) from 1993 to 2006, which was lower than 1.92% (95% CI, 1.11-2.73%) for females. In addition, we found that the top two age groups of HBsAg seroclearance for males were the 5-9 and 20-24 age groups and were the 1-4 and 5-9 age groups for females (see **Table 2**). Furthermore, we also estimated the sex-specific horizontal transmission rate$\beta_{a}^{g}(t)$, which was given by

$\beta_{a}^{m}(t)=\left(b_{1}^{m}\exp(-b_{2}^{m}(t-1992))+\left(1-b_{1}^{m}\right)\right)\beta_{a}^{m}(1992),$ (7)

$\beta_{a}^{f}(t)=\left(b_{1}^{f}\exp(-b_{2}^{f}(t-1992))+(1-b_{1}^{f})\right)\beta_{a}^{f}(1992),$ (8)

where

$b_{1}^{m}=0.6910, (95\%CI, \text{0.6494-0.7325})$, $b_{2}^{m}=0.5678, (95\%CI, \text{0.5434-0.5923})$;

$b_{1}^{f}=0.6043, (95\%CI, \text{0.5876-0.6209})$, $b_{2}^{f}=0.4088, (95\%CI, \text{0.3871-0.4305})$.

**4. Supplementary Tables**

**Table S1.** Parameter description for models (1) and (2)

| Parameters | Meanings | Values | References |
| --- | --- | --- | --- |
| $b(t)$ | Birth rate in year *t* | See Supplementary data.xlsx, column 1 | [10] |
| $k^{g}(t)$ | Proportion of sex-specific newborns at birth in year *t* | Figure S1a | [11, 12] |
| $v(t)$ | Vaccination coverage rate of newborns in year *t* | See Supplementary data.xlsx, column 2 | [13] |
| $m_{_{a}}^{g}$ | Age- and sex-specific mortality rates of HBV-related diseases | Figure S1b | [14] |
| $p$ | Vaccination protection rate per year | 0.85 (0.75-0.95) | [16] |
| $\varepsilon$ | Perinatal infection rate per year | 0.06 (0.03-0.09) | [16] |
| $\omega$ | Proportion of HBsAg positive mothers aged 20–49 years to the total prevalence of HBsAg | 0.8436 | [16] |
| $\mu_{_{a}}^{g}(t)$ | Age-specific and sex-specific death rates of non-HBV related diseases in year *t* | $\mu_{a}^{g}(t)=(d_{a}^{g}(t)N_{a}^{g}(t)-m_{a}^{g}C_{a}^{g}(t))/N_{a}^{g}(t)$ | [16] |
| $q_{p}$ | Proportion of acute HBV infections that became chronic during the delivery period | 0.90 | [23-25] |
| $d_{a}^{g}(t)$ | Age- and sex-specific total death rates in year *t* | Figure S1c and Figure S1d | [15, 16] |
|  | 1993 ≤ *t* ≤ 1997 | $d_{a}^{g}(t)=d_{a}^{g}(1995)$ |  |
|  | 1998 ≤ *t* ≤ 2002 | $d_{a}^{g}(t)=d_{a}^{g}(2000)$ |  |
|  | 2003 ≤ *t* ≤ 2007 | $d_{a}^{g}(t)=d_{a}^{g}(2005)$ |  |
|  | 2008 ≤ *t* ≤ 2012 | $d_{a}^{g}(t)=d_{a}^{g}(2010)$ |  |
|  | 2013 ≤ *t* ≤ 2035 | $d_{a}^{g}(t)=d_{a}^{g}(2015)$ |  |
| $\theta_{a}(t)$ | Catch-up vaccination coverage rate for adolescents aged 8-15 years during the years 2009-2011 | 0.95 | [26] |
| $q{}_{a}$ | Age-specific proportion of acute HBV infections that became chronic per year |  | [23-25] |
|  | 0 ≤ *a*< 1 | 0.30 |  |
|  | 1≤ *a*≤ 4 | 0.25 |  |
|  | 5 ≤ *a* ≤ 15 | 0.06 |  |
|  | 16 ≤ *a* ≤ 100 | 0.03 |  |
| $\beta_{a}^{m}(1992)$ | Horizontal transmission rate for males in 1992 | Figure S1e | [16] |
| $\beta_{a}^{f}(1992)$ | Horizontal transmission rate for females in 1992 | Figure S1f | [16] |
| $\beta_{m}$ | Sexual transmission probability of HBV per-partnership for males per year | 0.024 | [27] |
| $\beta_{f}$ | Sexual transmission probability of HBV per-partnership for females per year | 0.009 | [27] |

**Table S2.** Estimated average numbers of sexual partners per year in each age group.

| Age group  (years) | Male | | Female | |
| --- | --- | --- | --- | --- |
|  | **sexual partners** | **95% CI** | **sexual partners** | **95%CI** |
| 20-24 | 1.62 | (1.60, 1.64) | 1.72 | (1.71, 1.74) |
| 25-29 | 1.76 | (1.74, 1.79) | 1.83 | (1.80, 1.85) |
| 30-34 | 1.92 | (1.90, 1.95) | 2.12 | (2.11, 2.14) |
| 35-39 | 1.59 | (1.57, 1.61) | 1.70 | (1.69, 1.72) |
| 40-49 | 1.33 | (1.31, 1.34) | 1.39 | (1.38, 1.40) |
| 50-60 | 1.11 | (1.10-1.12) | 1.21 | (1.18, 1.24) |

**Table S3.** Sensitivity analysis results for HBV infections.

| Parameters | | Number of chronic HBV infections in males in 2035 | Number of chronic HBV infections in females in 2035 | Number of acute HBV infections in males in 2035 | Number of acute HBV infections in females in 2035 | Number of HBV-related deaths of males in 2035 | Number of HBV-related deaths of females in 2035 |
| --- | --- | --- | --- | --- | --- | --- | --- |
| Current practice | | 22806441 | 16014056 | 307571 | 326930 | 306624 | 140103 |
| $\beta_{a}^{m}(1992)$ | **The upper limit value** | 24128523 | 16050250 | 386200 | 339514 | 311334 | 140166 |
|  | **Increase from current practice (%)** | 5.80% | 0.23% | 25.56% | 3.85% | 1.54% | 0.04% |
|  | **The lower limit value** | 21818721 | 15986903 | 246852 | 317498 | 303116 | 140056 |
|  | **Decrease from current practice (%)** | 4.33% | 0.17% | 19.74% | 2.89% | 1.14% | 0.03% |
| $\beta_{a}^{f}(1992)$ | **The upper limit value** | 22852209 | 16919379 | 319103 | 409093 | 306750 | 141699 |
|  | **Increase from current practice (%)** | 0.20% | 5.65% | 3.75% | 25.13% | 0.04% | 1.14% |
|  | **The lower limit value** | 22773489 | 15362620 | 299289 | 262941 | 306533 | 138914 |
|  | **Decrease from current practice (%)** | 0.14% | 4.07% | 2.69% | 19.57% | 0.02% | 0.85% |
| Vaccination protection rate | **0.95** | 22101281 | 15560323 | 211263 | 225501 | 306012 | 139924 |
|  | **Decrease from current practice (%)** | 3.09% | 2.83% | 31.31% | 31.02% | 0.20% | 0.13% |
|  | **0.75** | 23528793 | 16479627 | 409035 | 433767 | 307241 | 140284 |
|  | **Increase from current practice (%)** | 3.17% | 2.91% | 32.99% | 32.68% | 0.20% | 0.13% |
| Perinatal infection rate | **0.09** | 23167822 | 16156958 | 316016 | 335122 | 307000 | 140167 |
|  | **Increase from current practice (%)** | 1.58% | 0.89% | 2.75% | 2.51% | 0.12% | 0.05% |
|  | **0.03** | 22447303 | 15872317 | 299366 | 318941 | 306247 | 140039 |
|  | **Decrease from current practice (%)** | 1.57% | 0.89% | 2.67% | 2.44% | 0.12% | 0.05% |

**5. Supplementary Figures**

**
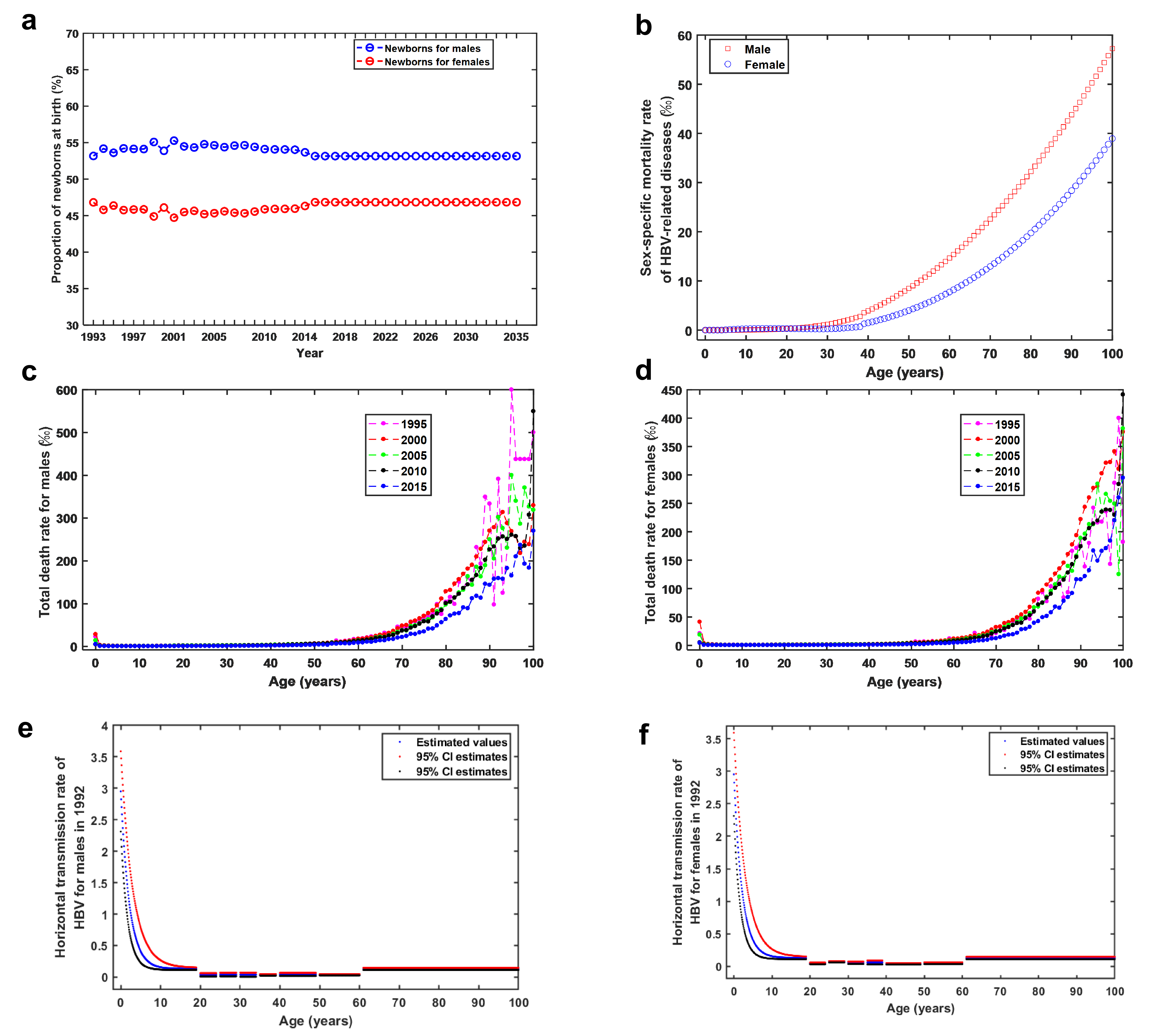
**

**Figure S1. Model input parameters.** (a) Proportion of sex-specific newborns at birth in year t. (b) Age-specific and sex-specific mortality rates of HBV-related diseases. (c) Age-specific total death rate for males in 1995, 2000, 2005, 2010 and 2015. (d) Age-specific total death rate for females in 1995, 2000, 2005, 2010 and 2015. (e) The age-specific horizontal transmission rate for males in 1992 and its 95% confidence intervals. (f) The age-specific horizontal transmission rate for females in 1992 and its 95% confidence intervals.

**
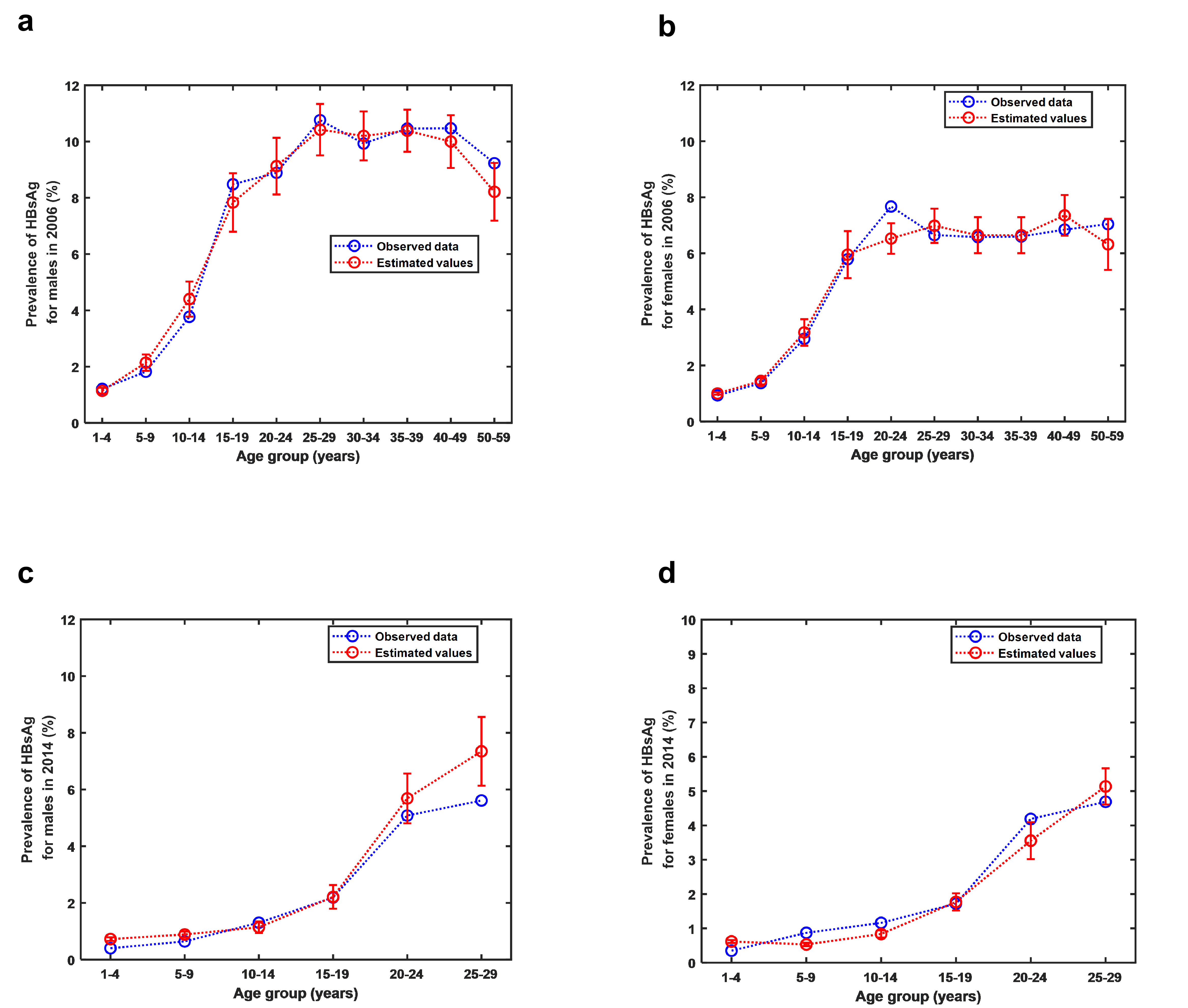
**

**Figure S2. Comparison of estimated age- and sex-specific prevalence of HBsAg with the observed data.** (a) In males in 2006. (b) In females in 2006. (c) In males in 2014. (d) In females in 2014.


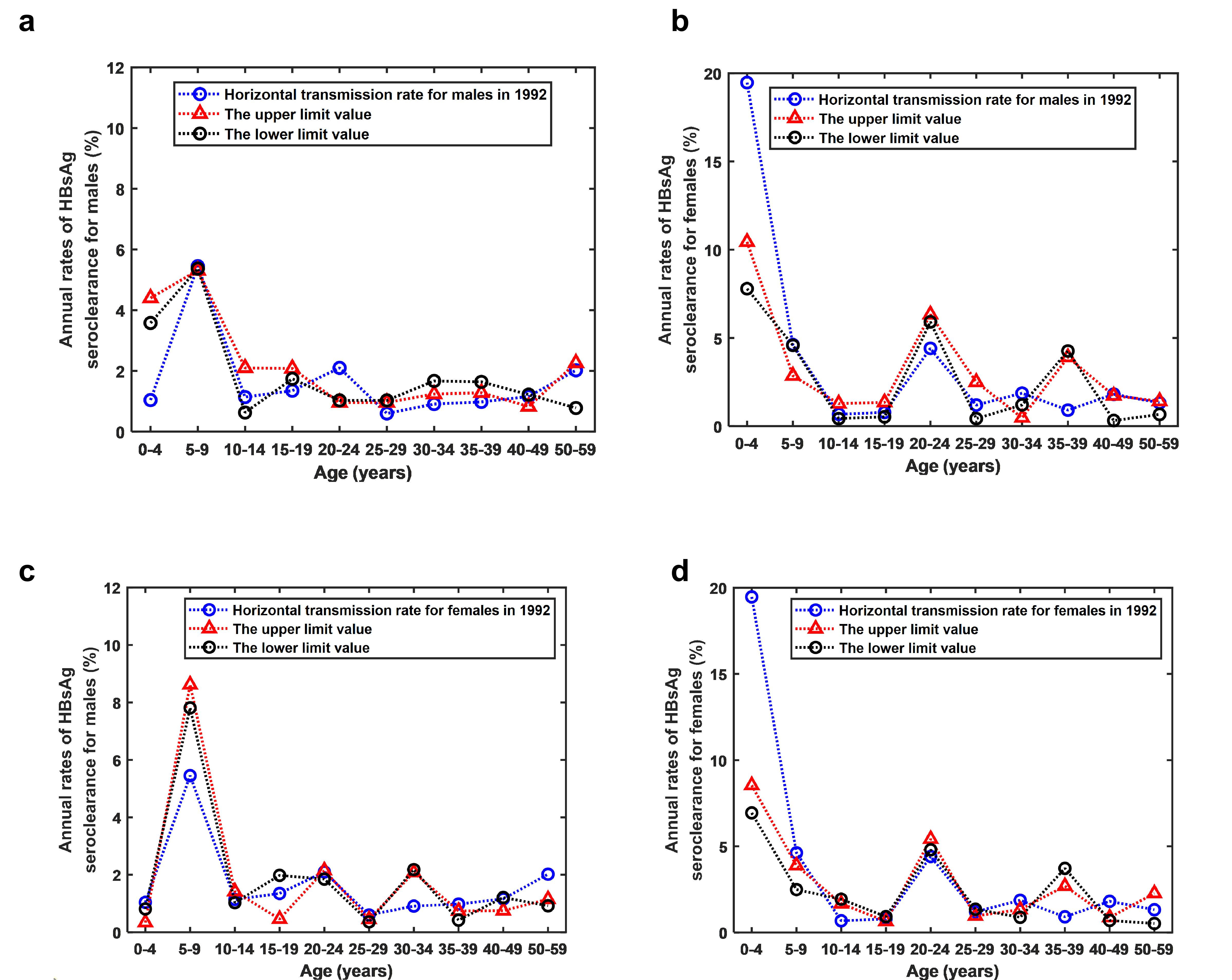


**Figure S3. Effect of sex-specific horizontal transmission rate on the sex-specific annual rate of HBsAg seroclearance.** (a) The effect of the horizontal transmission rate for males in 1992 on the annual rate of HBsAg seroclearance for males. (b) The effect of the horizontal transmission rate for males in 1992 on the annual rate of HBsAg seroclearance for females. (c) The effect of the horizontal transmission rate for females in 1992 on the annual rate of HBsAg seroclearance for males. (d) The effect of the horizontal transmission rate for females in 1992 on the annual rate of HBsAg seroclearance for females.


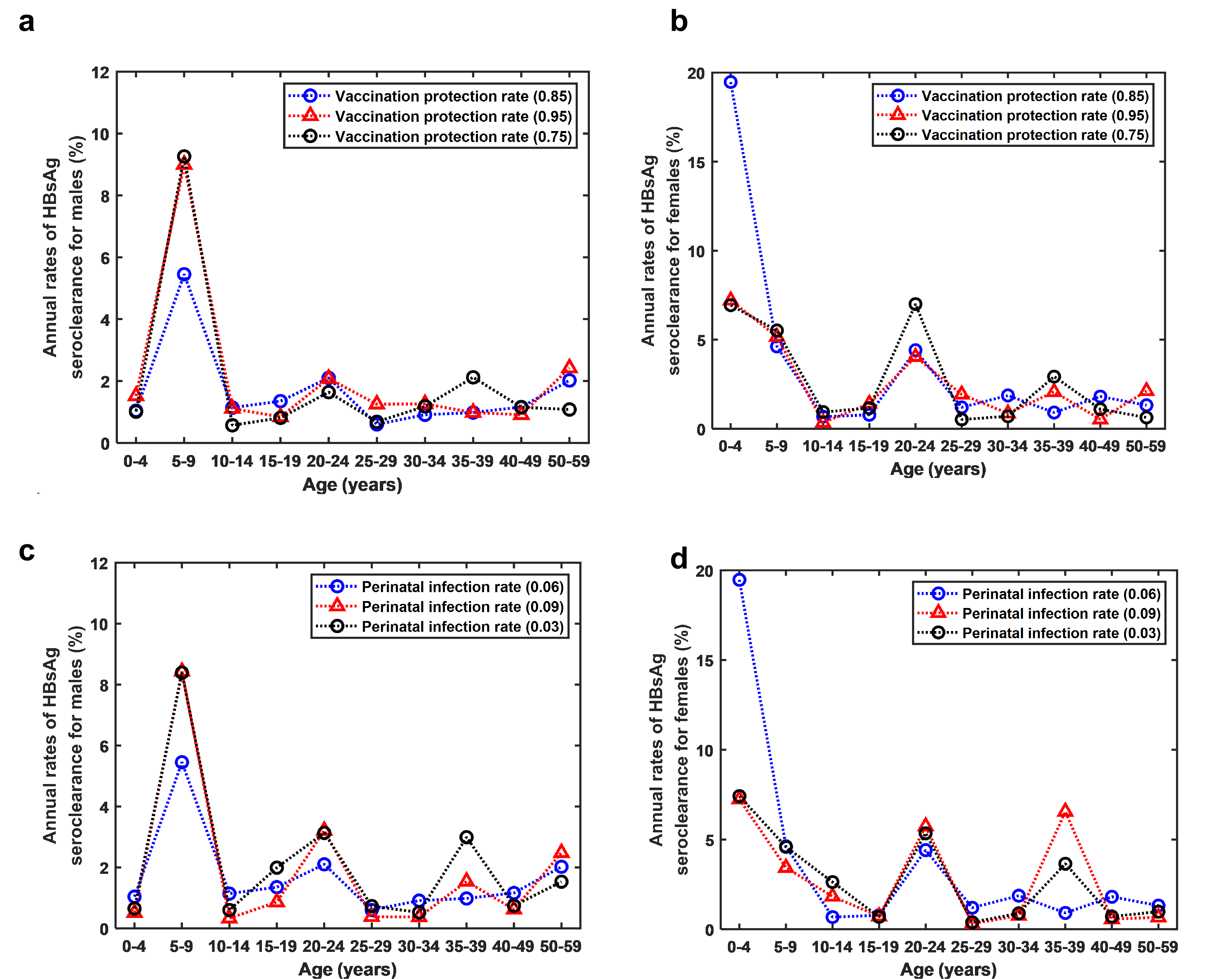


**Figure S4. Impact of vaccination protection rate and perinatal infection rate on the sex-specific annual rate of HBsAg seroclearance.** (a) The effect of the vaccination protection rate on the annual rate of HBsAg seroclearance for males. (b) The effect of the vaccination protection rate on the annual rate of HBsAg seroclearance for females. (c) The effect of the perinatal infection rate on the annual rate of HBsAg seroclearance for males. (d) The effect of the perinatal infection rate on the annual rate of HBsAg seroclearance for females.

**References**

1. Liang X, Bi S, Yang W, et al. Epidemiological serosurvey of hepatitis B in China-declining HBV prevalence due to hepatitis B vaccination. Vaccine, 2009; 27: 6550-6557.
2. Xia GL, Liu CB, Cao HL, et al. Prevalence of hepatitis B and C virus infections in the general Chinese population. Results from a nationwide cross-sectional seroepidemiologic study of hepatitis A, B, C, D, and E virus infections in China, 1992. International Hepatology Communications, 1996, 5: 62-73.
3. Cui F, Lipin Shen LL, Wang H, et al. Prevention of chronic hepatitis B after 3 decades of escalating vaccination policy, China. Emerging Infectious Diseases, 2017; 23: 765.
4. Ganem D, Prince AM. Hepatitis B virus infection—natural history and clinical consequences. New England Journal of Medicine, 2004; 350: 1118-1129.
5. McMahon BJ. The natural history of chronic hepatitis B virus infection. Hepatology, 2009; 49: S45-S55.
6. Lu FM, Li T, Liu S, et al. Epidemiology and prevention of hepatitis B virus infection in China. Journal of Viral Hepatitis, 2010; 17: 4-9.
7. Liu J, Fan D. Hepatitis B in China. The Lancet, 2007, 369: 1582-1583.
8. Lee WM. Hepatitis B virus infection. New England Journal of Medicine, 1997; 337: 1733-1745.
9. Keeling MJ, Rohani P. Modeling infectious diseases in humans and animals. Princeton University Press, 2011.
10. National Bureau of Statistics of China. The birth rate per year. http://data.stats.gov.cn/easyquery.htm?cn=C01.
11. Tang, ZY. Research on the Sex Ratio of Birth Population in China. China Yan Shi Press, Beijing, 2008. (in Chinese).
12. National Bureau of Statistics of China. National economy and society developed statistical bulletin (from 2006-2015).
13. Liang X, Bi S, Yang W, et al. Evaluation of the impact of hepatitis B vaccination among children born during 1992–2005 in China. The Journal of Infectious Diseases, 2009; 200: 39-47.
14. Goldstein ST, Zhou F, Hadler SC, et al. A mathematical model to estimate global hepatitis B disease burden and vaccination impact. International Journal of Epidemiology, 2005; 34: 1329-1339.
15. China Population and Employment Statistics Yearbook, Department of Population and Employment Statistics, National Bureau of Statistics. Beijing: China Statistics Press (from 2008-2017).
16. Zu J, Zhuang G, Liang P, et al. Estimating age-related incidence of HBsAg seroclearance in chronic hepatitis B virus infections of China by using a dynamic compartmental model. Scientific Reports, 2017; 7: 2912.
17. China Population Statistics Yearbook, Department of Population and Employment Statistics, National Bureau of Statistics. Beijing: China Statistics Press (from 1993-2007).
18. Anderson RM, May RM. Directly transmitted infectious diseases: control by vaccination. Science, 1982, 215: 1053-1060.
19. Pan SM. Sexual behavior and relation in contemporary China. Social Sciences Academic Press, 2004. (in Chinese).
20. Keeffe EB. Clinical approach to viral hepatitis in homosexual men. Medical Clinics of North America, 1986, 70: 567-586.
21. Kao JH, Chen DS. Global control of hepatitis B virus infection. The Lancet infectious diseases, 2002, 2: 395-403.
22. Lai CL, Yuen MF. Prevention of hepatitis B virus–related hepatocellular carcinoma with antiviral therapy. Hepatology, 2013, 57: 399-408.
23. Liaw YF, Chu CM. Hepatitis B virus infection. Lancet, 2009, 373: 582-592.
24. Hsu HY, Chang MH, Hsieh KH, et al. Cellular immune response to HBcAg in mother‐to‐infant transmission of hepatitis B virus. Hepatology, 1992, 15: 770-776.
25. Hong M, Sandalova E, Low D, et al. Trained immunity in newborn infants of HBV-infected mothers. Nature Communications, 2015, 6: 6588.
26. Liao X, Liang Z. Strategy vaccination against Hepatitis B in China. Human Vaccines and Immunotherapeutics, 2015, 11: 1534-1539.
27. Lu XW, Liao Q. Investigation on the spread of hepatitis B virus between husband and wife. Shenzhen Journal of Integrated Traditional Chinese and Western Medicine, 2018, 02-0028-02. (in Chinese).
